# Effect of different levels of acute hypoxia on subsequent oral glucose tolerance in males with overweight: A balanced cross‐over pilot feasibility study

**DOI:** 10.14814/phy2.15623

**Published:** 2023-05-05

**Authors:** Jo Corbett, Michael J. Tipton, Maria Perissiou, Thomas James, John S. Young, Alexander Newman, Michael Cummings, Hugh Montgomery, Michael P. W. Grocott, Anthony I. Shepherd

**Affiliations:** ^1^ Extreme Environments Laboratory, School of Sport, Health and Exercise Science University of Portsmouth Portsmouth UK; ^2^ Clinical, Health and Rehabilitation Team, School of Sport, Health and Exercise Science University of Portsmouth Portsmouth UK; ^3^ National Horizons Centre Teesside University Middlesbrough UK; ^4^ School of Health and Life Sciences Teesside University Middlesbrough UK; ^5^ Diabetes and Endocrinology Department Portsmouth Hospitals University NHS Trust Portsmouth UK; ^6^ Centre for Sport Exercise and Health, Dept Medicine University College London London UK; ^7^ Perioperative and Critical Care Theme, NIHR Southampton Biomedical Research Centre University Hospital Southampton/University of Southampton Southampton UK

**Keywords:** altitude, diabetes, metabolic disease, environmental stress, reduced oxygen

## Abstract

Previous research has shown that ≤60 min hypoxic exposure improves subsequent glycaemic control, but the optimal level of hypoxia is unknown and data are lacking from individuals with overweight. We undertook a cross‐over pilot feasibility study investigating the effect of 60‐min prior resting exposure to different inspired oxygen fractions (CON F_I_O_2_ = 0.209; HIGH F_I_O_2_ = 0.155; VHIGH F_I_O_2_ = 0.125) on glycaemic control, insulin sensitivity, and oxidative stress during a subsequent oral glucose tolerance test (OGTT) in males with overweight (mean (SD) BMI = 27.6 (1.3) kg/m^2^; *n* = 12). Feasibility was defined by exceeding predefined withdrawal criteria for peripheral blood oxygen saturation (SpO_2_), partial pressure of end‐tidal oxygen or carbon dioxide and acute mountain sickness (AMS), and dyspnoea symptomology. Hypoxia reduced SpO_2_ in a stepwise manner (CON = 97(1)%; HIGH = 91(1)%; VHIGH = 81(3)%, *p* < 0.001), but did not affect peak plasma glucose concentration (CON = 7.5(1.8) mmol∙L^−1^; HIGH = 7.7(1.1) mmol∙L^−1^; VHIGH = 7.7(1.1) mmol∙L^−1^; *p* = 0.777; *η* ^2^ = 0.013), plasma glucose area under the curve, insulin sensitivity, or metabolic clearance rate of glucose (*p* > 0.05). We observed no between‐conditions differences in oxidative stress (*p* > 0.05), but dyspnoea and AMS symptoms increased in VHIGH (*p* < 0.05), with one participant meeting the withdrawal criteria. Acute HIGH or VHIGH exposure prior to an OGTT does not influence glucose homeostasis in males with overweight, but VHIGH is associated with adverse symptomology and reduced feasibility.

## 1 INTRODUCTION

Acute exposure to hypoxia can positively influence glycaemic control in the subsequent hours. For example, Duennwald et al. (2013) reported that, compared to normoxia, a 1‐h bout of intermittent hypoxia (5 × 6‐min breathing a fractional inspired oxygen concentration (F_I_O_2_) of 0.13, separated by 6‐min recovery) lowered blood glucose concentration ([glucose]) and diminished the glycaemic response to a subsequent meal in individuals with type 2 diabetes mellitus (T2DM). Likewise, Mackenzie et al. (2011) demonstrated that when individuals with T2DM breathed an F_I_O_2_ of 0.146 for 60 min, blood [glucose] and blood insulin area under the curve (AUC) were reduced, and insulin sensitivity improved, during a subsequent intravenous glucose tolerance test. In men with obesity (mean(SD) body mass index (BMI) = 32.7(1.3) kg/m^2^), a more prolonged intervention consisting of ten nights of moderate normobaric hypoxia (F_I_O_2_ = 0.15) reduced fasting blood [glucose] and improved insulin sensitivity (Lecoultre et al., 2013). However, this type of approach may be logistically challenging and it is currently unclear whether a shorter duration of intervention is effective in individuals with overweight, as has been shown for individuals with T2DM. Moreover, whilst a recent systematic‐review concluded that passive hypoxia may improve glucose homeostasis in metabolically compromised (overweight/obese/metabolic syndrome/T2DM) individuals (van Hulten et al., 2021), it was acknowledged that the data were equivocal, possibly due to methodological heterogeneity in a number of factors, including the hypoxic stressor.

Although positive effects on glycaemic control have been demonstrated with repeated brief exposures to a low F_I_O_2_ (e.g., 0.12–0.13 [Duennwald et al., 2013; Serebrovska et al., 2017]), other research suggests that higher levels of hypoxia, such as those simulating obstructive sleep apnoea (Louis & Punjabi, 2009), or more prolonged exposure to high altitudes >4000 m (Braun et al., 2001; Siervo et al., 2014), impair glycaemic control (see Mackenzie & Watt, 2016, for review). High levels of hypoxic stress are also associated with increased inflammatory markers (e.g., interleukin‐6 [IL‐6]) and oxidative stress markers (e.g., advanced oxidation protein product [AOPP]) (Hartmann et al., 2000; Ribon et al., 2016; Siervo et al., 2014), which are implicated in the pathophysiology of chronic diseases (Wu et al., 2014), as well as adverse symptomology, including headache, nausea, and dyspnoea (Roach et al., 2018). High body mass index may also be associated with a reduced SpO_2_ (Kapur et al., 2013) and increased acute mountain sickness symptoms with exposure to a hypoxic environment (Wu et al., 2012); these factors may compromise the feasibility of any acute hypoxic intervention, particularly in a cohort with overweight. Optimizing the hypoxic stress is, therefore, important for informing future research examining therapeutic effects of hypoxia in metabolically compromised individuals. Yet, to date, no studies have compared the impact of different levels of acute hypoxia on subsequent glycaemic control.

Accordingly, we performed a pilot‐feasibility study examining the short‐term effects of different levels of acute hypoxia on glucose tolerance, insulin sensitivity, markers of inflammation and oxidative stress, and feasibility in males with overweight—a group predisposed to impaired glucose homeostasis (Wannamethee & Shaper, 1999).

## 2 METHOD

### 2.1 Design

A single‐blind, balanced cross‐over design consisting of three conditions: a normobaric‐normoxic control (CON; F_I_O_2_ = 0.209) and two normobaric‐hypoxic conditions equivalent to a simulated altitude of ~2500 m, i.e., ‘high’ altitude (HIGH; target F_I_O_2_ = 0.155), and ~ 4000 m, i.e., ‘very‐high’ altitude (VHIGH; target F_I_O_2_ = 0.125) (Imray et al., 2011). Participants were allocated to one of the six possible condition combinations by order of study enrolment.

### 2.2 Participants

To balance the three conditions, a convenience sample of 12 males with overweight (BMI 25 to 30.0 kg/m^2^) was recruited from the host university (mean (SD) age = 32(7) years; height = 1.82(0.08) m; mass = 91.8(10.1) kg; BMI = 27.6(1.3) kg/m^2^). Although intended as a pilot feasibility‐study, our sample size was in keeping with similar previous work that informed the study (Duennwald et al., 2013; Lecoultre et al., 2013; Mackenzie et al., 2011), with a power calculation indicating that this sample would enable detection an effect size (η^2^) of 0.14 for a given outcome variable using repeated measures ANOVA (alpha = 0.05; power = 0.8; correlation among reported measures = 0.5; non‐sphericity correction = 1; G*power 3.1.9.7). All participants were free from metabolic disease including T2DM (glycated hemoglobin (HbA1c) = 34(4) mmol/mol), thalassemia, and haemoglobinopathies, were not taking any medications and had normal resting lung function (FVC <80% of predicted FVC, FEV_1_ < 70% of FVC), ECG, and blood pressure.

### 2.3 Materials and methods

Participants reported to the laboratory at 0730 hours on test days following a 12‐h overnight fast and in a hydrated state, having only consumed water and having abstained from alcohol and strenuous exercise in the previous 24 h. Participants rested in a semi‐recumbent position and a venous cannula was inserted into a forearm vein. Thereafter, following a 15‐min rest period, baseline measurements were obtained and the participant donned an oronasal mask delivering the required F_I_O_2_ (Cloud 9, Sporting Edge, Basingstoke, UK) and connected to a metabolic cart (Quark CPET, Cosmed, Italy) calibrated using known concentrations of O_2_, CO_2_, and N_2_ gas, and a 3‐L air syringe (Hans Rudolph, US). Participants were blind to the F_I_O_2_ on each occasion with CON serving as a sham control condition. Pulmonary gas exchange and ventilation were averaged into 60‐s time bins. Peripheral oxygen saturation was measured by fingertip pulse oximetry (Nonin 7500, US) interfaced with a data acquisition system (Powerlab, AD Instruments, New Zealand) and heart rate using short range telemetry (Polar Electro, Finland). Participants were withdrawn if their peripheral blood oxygen saturation (SpO_2_) fell below 65% for 15 continuous seconds, or if the partial pressure of end‐tidal oxygen (P_ET_O_2_) fell below 45 mmHg, or the partial pressure of end‐tidal carbon dioxide (P_ET_CO_2_) fell below 25 mmHg, for three consecutive breaths. Preliminary data collection indicated that, for most participants, the lower F_I_O_2_ would approximate the exposure limits permitted within our laboratory.

Informed by previous studies showing beneficial effects of acute hypoxia on subsequent glycaemic control (Duennwald et al., 2013; Mackenzie et al., 2011), participants were exposed to hypoxia for 60‐min. After exposure to the hypoxic environment, resting blood pressure (Omron, Japan) was measured and the Lake Louise Acute Mountain Sickness (AMS) questionnaire (Roach et al., 2018) and modified Borg dyspnoea scales (Mahler & Horowitz, 1994) were administered. The participant then ingested a 75 g glucose solution (PenLan Healthcare, Rapilose, UK) and the 120‐min oral glucose tolerance test (OGTT) commenced, with the participant now breathing (normoxic) room air. Blood samples were drawn into 6–8 mL EDTA and fluoride oxalate vacutainers and centrifuged (4500 g for 10 min at 4°C) to separate plasma. Blood samples were obtained before and after hypoxic exposure and at 30‐min intervals until completion of the OGTT; hunger (100‐mm visual analogue scale) was measured at the same time points. Plasma was stored at −80°C until analysis. Plasma [glucose] was analyzed using an automated analyzer (C‐Line, Biosen, UK) and plasma insulin concentration ([insulin]), oxidative stress (AOPP), and inflammation (IL‐6) using ELISA kits (DY8056‐05, BioTechne, UK; ab242295, Abcam, UK; DY206‐05, BioTechne, UK, respectively) read using a plate reader (SpectraMax i3x, Molecular Devices, UK).

### 2.4 Analysis

Statistical analyses were undertaken using SPSS Version 28 (IBM, USA), and significance was set a priori at P ≤ 0.05. Data are presented mean (SD), with the exception of the Lake Louise questionnaire and dyspnoea scale data, which are presented as median(range). Peak and AUC (trapezoid method) were calculated for [glucose] and AOPP to provide an index of glucose homeostasis and a marker of oxidative stress, respectively, following the hypoxic exposure. Post‐prandial insulin sensitivity and metabolic clearance rate of glucose (a maker of insulin sensitivity) were calculated according to validated equations incorporating demographic data (age and BMI) and on the basis of [glucose] and [insulin] samples at 0, 60, and 120 min of the OGTT (Stumvoll et al., 2001). Peak [glucose] and insulin sensitivity were primary outcome measures. S_P_O_2_ and heart rate was averaged over the 1‐h normobaric‐hypoxic exposures and CON equivalent; physiologically implausible data (e.g., sensors falling off) were removed. Energy expenditure and substrate utilization were calculated during the OGTT using indirect respiratory calorimetry, and hunger scores were summed over the OGTT period to create a single hunger score. Criteria for feasibility were (i) withdrawal from the study due to physiological perturbation exceeding any of the study withdrawal criteria for SpO_2_, P_ET_O_2_, or P_ET_CO_2_ or (ii) symptoms attributable to acute hypoxia (AMS and dyspnoea). Between‐conditions differences were examined using one‐way RM‐ANOVA, with the exception of AMS symptoms and perceived dyspnoea, which were analyzed using Friedman's test; if the RM‐ANOVA violated the assumption of sphericity, the Greenhouse–Geisser statistic was employed. The effect size for RM ANOVA is reported as *η* ^2^, and post‐hoc analysis of significant RM‐ANOVA and Friedman's test effects were undertaken using (least significant difference) pairwise comparisons and Wilcoxon's signed ranks test, respectively.

## 3 RESULTS

**FIGURE 1:**
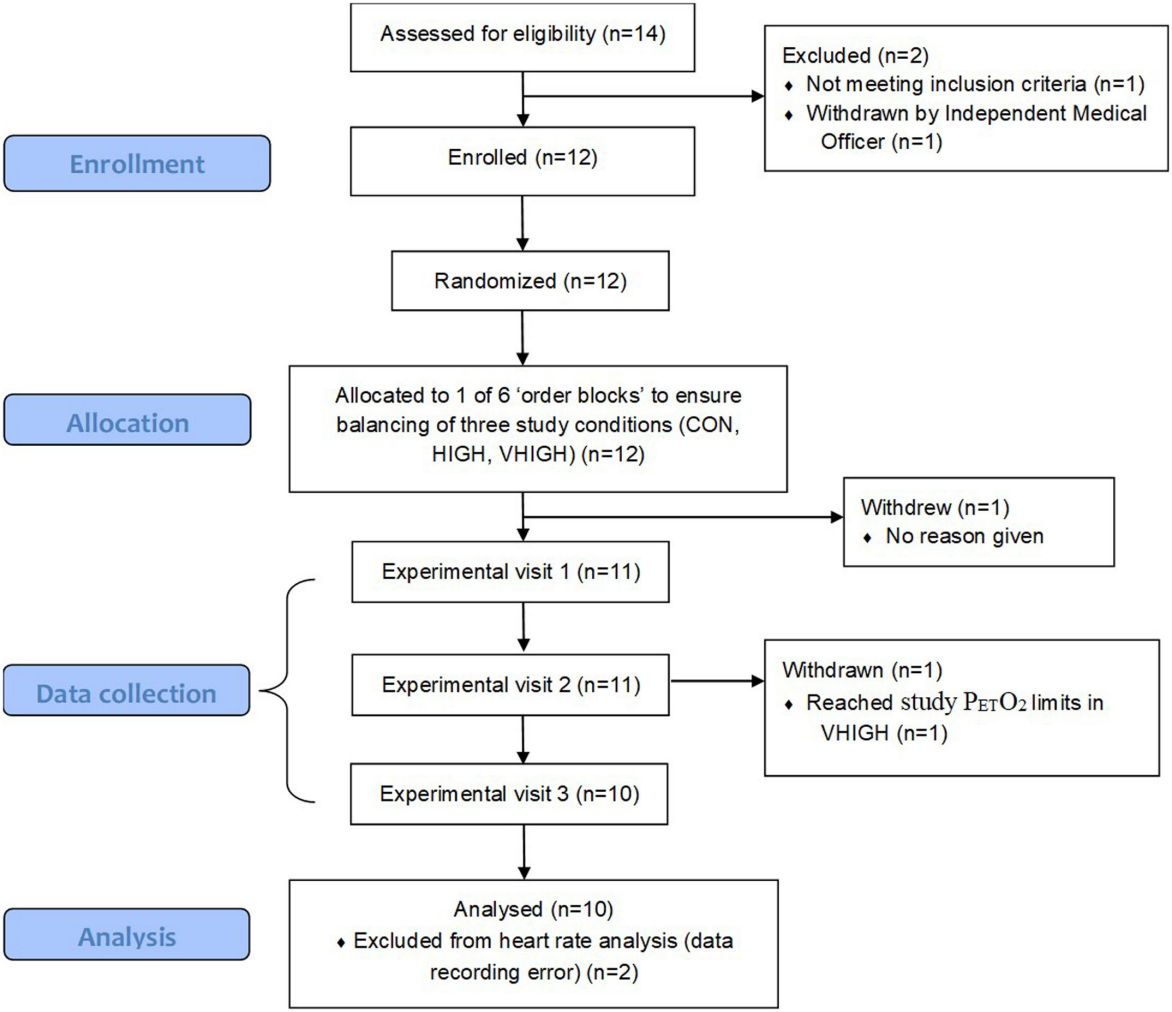
Consort flow diagram.

One participant was unable to complete the experimental trials due to reaching the study P_ET_O_2_ withdrawal criteria in VHIGH and one participant withdrew after providing their written informed consent (see Figure 1). Therefore, data are for 10 participants, unless otherwise stated. F_I_O_2_ measured during the hypoxic trials was 0.154(0.02) and 0.126(0.05) for the HIGH and VHIGH trials, respectively. SpO_2_ differed between all conditions (CON = 97(1)%; HIGH = 91(1)%; VHIGH = 81(3)%; *p* < 0.001, *η* ^2^ = 0.957) being highest in CON and lowest in VHIGH (all *p* < 0.001).

**FIGURE 2:**
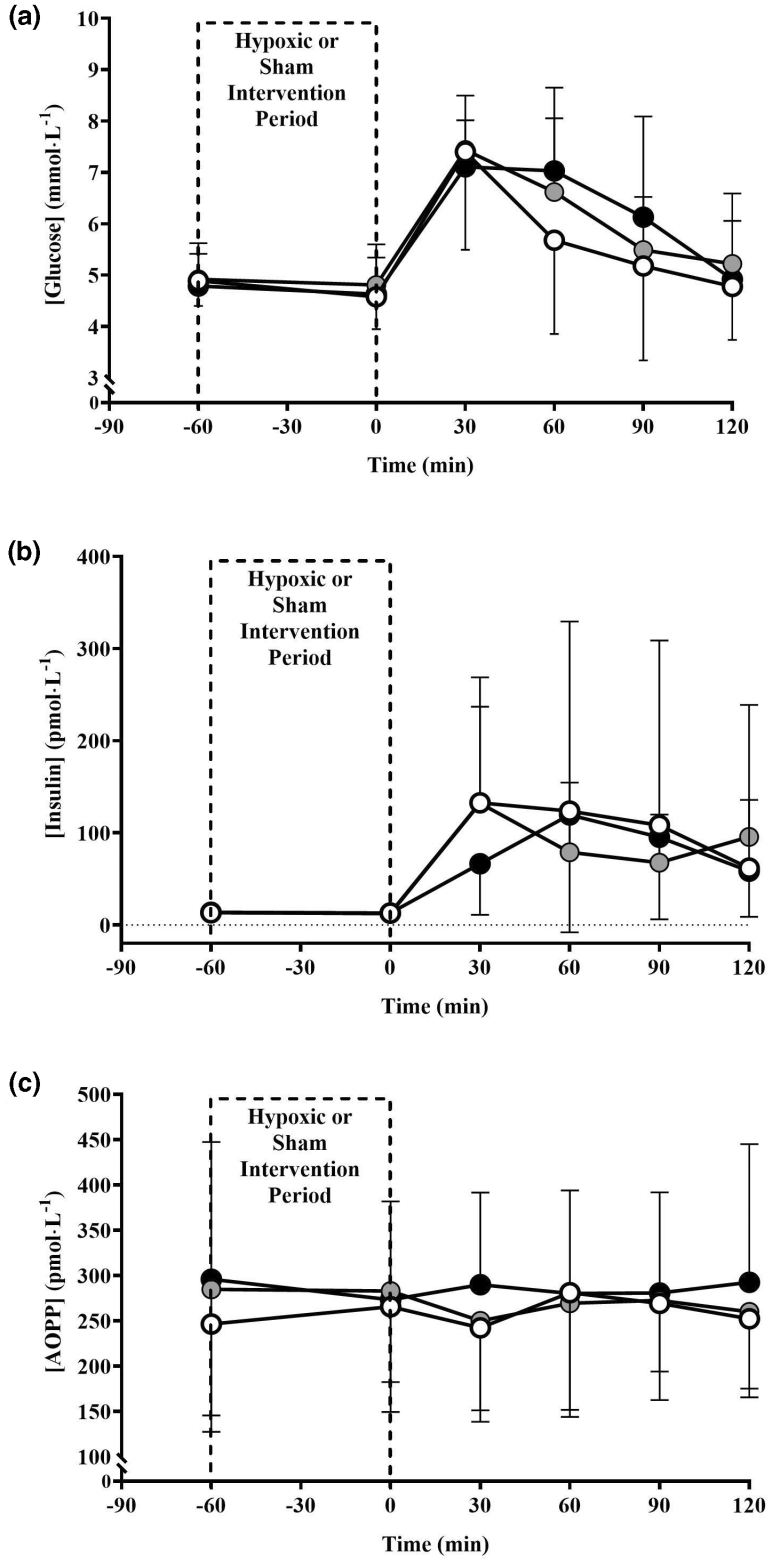
(a) Plasma glucose concentration ([glucose]), (b) plasma insulin concentration ([insulin]), and (c) plasma advanced oxidation protein product concentration ([AOPP]), pre and post 60 min of normoxia F_I_O_2_ = 0.209 (CON; open circles) or normobaric hypoxia at an F_I_O_2_ of 0.155 (HIGH; gray circles), or 0.125 (VHIGH; black circles) and during a subsequent 120‐min OGTT.

**FIGURE 3:**
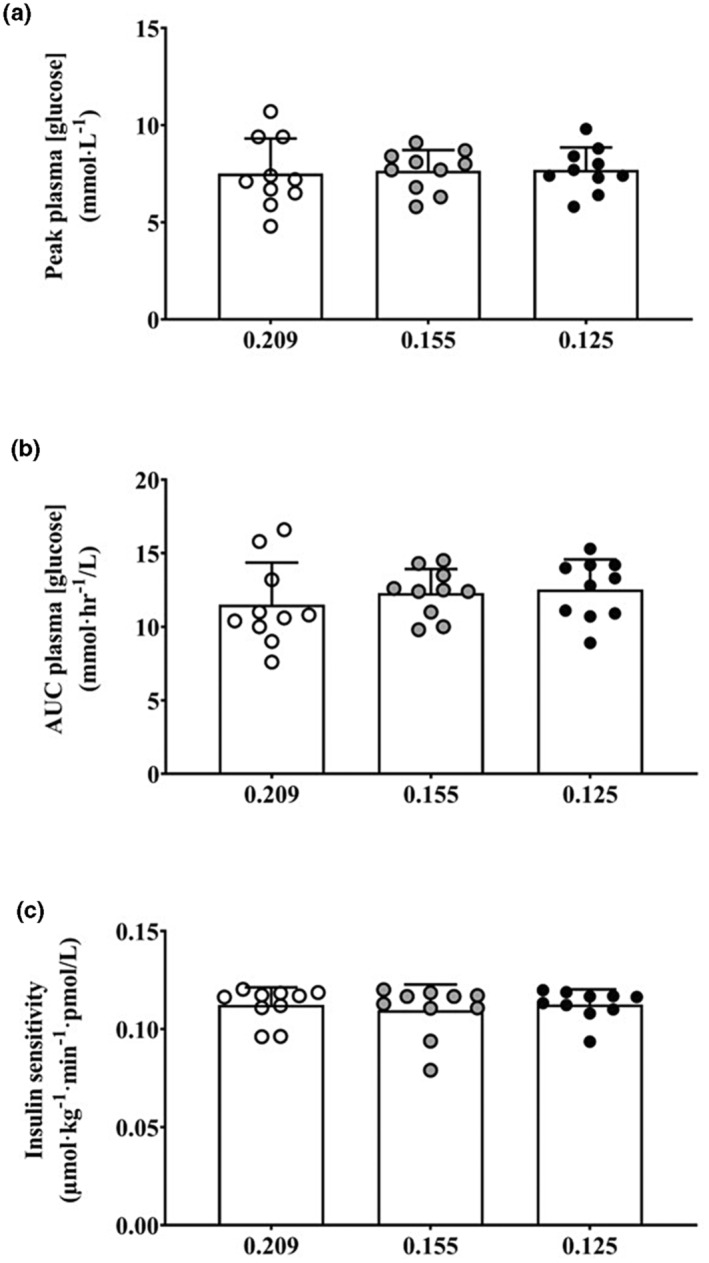
(a) Peak plasma glucose concentration ([glucose]), (b) plasma [glucose] area under the curve, and (c) insulin sensitivity during a 120‐min OGTT undertaken after 60 min of normoxia, F_I_O_2_ = 0.209 (CON; open circles), or normobaric hypoxia at an F_I_O_2_ of 0.155 (HIGH; gray circles) or 0.125 (VHIGH; black circles).

Plasma [glucose] and [insulin] before and after the hypoxic intervention period, and during the subsequent OGTT, are shown in Figure 2. Neither peak [glucose] (CON = 7.5(1.8) mmol∙L^−1^; HIGH = 7.7(1.1) mmol∙L^−1^; VHIGH = 7.7(1.1) mmol∙L^−1^; *p* = 0.777, *η* ^2^ = 0.013) during the OGTT nor the area under the curve (CON = 11.5(2.9) mmol∙h^−1^/L; HIGH = 12.3(1.6) mmol∙h^−1^/L; VHIGH = 12.5(2.0) mmol∙h^−1^/L; *p* = 0.241, *η* ^2^ = 0.146) differed between conditions (see Figure 3). Likewise, insulin sensitivity (CON = 0.11(0.01) μmol∙kg^−1^∙min^−1^∙pmol/L; HIGH = 0.11(0.01) μmol∙kg^−1^∙min^−1^∙pmol/L; VHIGH = 0.11(0.01) μmol∙kg^−1^∙min^−1^∙pmol/L; *p* = 0.351, *η* ^2^ = 0.099) (see Figure 3) and metabolic clearance rate of glucose (CON = 9.6(0.6) mL∙kg^−1^∙min^−1^; HIGH = 9.3(1.1) mL∙kg^−1^∙min^−1^; VHIGH = 9.6(0.7) mL∙kg^−1^∙min^−1^; *p* = 0.256, *η* ^2^ = 0.142) were unaffected by the F_I_O_2_. Oxidative stress (plasma [AOPP]) before and after the hypoxic intervention period, and during the subsequent OGTT, is shown in Figure 2. Peak oxidative stress (peak [AOPP]: CON = 320(125) pmol/L; HIGH = 335(98) pmol/L; VHIGH 353(119) pmol/L; *p* = 0.621, *η* ^2^ = 0.051) and accumulated oxidative stress (AUC [AOPP]: CON = 526(202) pmol∙h^−1^/L; HIGH = 532(176) pmol∙h^−1^/L; VHIGH 567(203) pmol∙h^−1^/L; *p* = 0.606, *η* ^2^ = 0.054) did not differ between the conditions, whereas IL‐6 remained below the detectable limits of the assay (9.4 pg∙mL^−1^).

Total energy expenditure during the OGTT differed between conditions (CON = 256(39) kcal; HIGH = 235(38) kcal; VHIGH = 247(32) kcal; *p* = 0.011, *η* ^2^ = 0.394), being lower in HIGH than VHIGH or CON (*p* < 0.05), although at an individual substrate level neither the total energy from fat (CON = 104(72) kcal; HIGH = 104(54) kcal; VHIGH = 126(46) kcal; *p* = 0.220, *η* ^2^ = 0.155) nor the total energy from carbohydrates (CON = 152(39) kcal; HIGH = 130(46) kcal; VHIGH = 121(30) kcal; *p* = 0.113, *η* ^2^ = 0.215) differed between conditions. Similarly, the average RER during the OGTT did not differ between conditions (CON = 0.90(0.08); HIGH = 0.87(0.06); VHIGH = 0.85(0.04); *p* = 0.092, *η* ^2^ = 0.233). Hunger score was unaffected by the hypoxic intervention (CON = 220(155) A.U; HIGH = 253(146) A.U; VHIGH = 240(123) A.U; *p* = 0.331, *η* ^2^ = 0.116).

Heart rate over the normobaric‐hypoxic exposure period differed between conditions (CON = 61(5) b∙min^−1^; HIGH = 64(6) b∙min^−1^; VHIGH = 73(10) b∙min^−1^; *p* = 0.008, *η* ^2^ = 0.629 (*n* = 8)), being higher in both HIGH and VHIGH than CON (both *p* < 0.01), and higher in VHIGH than HIGH (*p* < 0.05), but neither systolic blood pressure (CON = 129(10) mmHg; HIGH = 122(9) mmHg; VHIGH = 124(9) mmHg; *p* = 0.194, *η* ^2^ = 0.167), diastolic blood pressure (CON = 80(8) mmHg; HIGH = 77(6) mmHg; VHIGH = 78(8) mmHg; *p* = 0.574, *η* ^2^ = 0.060), nor mean arterial pressure (CON = 96(8) mmHg; HIGH = 92(7) mmHg; VHIGH = 93(6) mmHg; *p* = 0.263, *η* ^2^ = 0.138) differed.

There was a between‐conditions difference in AMS symptoms following the normobaric‐hypoxic exposures (CON = 0(0–2); HIGH = 0(0–1); VHIGH = 1(0–7); *p* = 0.009), which were greater in VHIGH compared to HIGH and CON (*p* < 0.05), with two participants meeting the criteria for AMS (Roach et al., 2018). Dyspnoea also differed between conditions (CON = 0(0–1); HIGH = 0(0–2); VHIGH = 1(0–4); *p* = 0.002), being higher in VHIGH than HIGH or CON (*p* < 0.05).

## 4 DISCUSSION

The aim of the present study was to assess the short‐term effects of different levels of acute hypoxia on subsequent glucose tolerance, insulin sensitivity, markers of inflammation and oxidative stress, and feasibility in males with overweight. Our main findings were that, following 60 min of breathing either of two hypoxic gas mixtures: (1) neither glucose tolerance, as assessed by peak [glucose] and [glucose] AUC, insulin sensitivity, nor metabolic clearance rate of glucose significantly differed across conditions; (2) markers of oxidative stress and inflammation were not elevated by the acute hypoxic exposures; and (3) feasibility was reduced at the lowest F_I_O_2_ (VHIGH).

Hypoxia has been shown to increase muscle glucose uptake through effects on insulin‐independent (Mackenzie & Watt, 2016) and insulin‐dependent mechanisms (Fisher et al., 2002) but is also associated with an augmented catecholamine release (Siervo et al., 2014) and accelerated rates of glycogenolysis (Clutter et al., 1988), although catecholamine changes may be limited during acute rather than chronic exposure to hypoxia (Rostrup, 1998). Thus, it is perhaps unsurprising that results from the studies examining the effect of hypoxia on glucose homeostasis are equivocal (van Hulten et al., 2021). Our data are in accordance with previous studies in healthy participants, such as that performed by Chan et al. (2021) who showed that an OGTT undertaken at an F_I_O_2_ of 0.148 did not differ from a normoxic control, and Morishima and Goto (2014) who showed no effect of 7‐h normobaric hypoxia (F_I_O_2_ = 0.155) on resting postprandial glucose regulation. They differ, however, from those showing beneficial effects of acute hypoxia on subsequent glycaemic control in metabolically compromised individuals (Duennwald et al., 2013; Mackenzie et al., 2011). Nevertheless, it is important to acknowledge that our study was designed as a pilot feasibility study to inform future research, and although our sample size was similar to other studies demonstrating an effect of acute exposure to hypoxia on subsequent glycaemic control (Duennwald et al., 2013; Lecoultre et al., 2013; Mackenzie et al., 2011), the medium effect size for insulin sensitivity (*η* ^2^ = 0.099) indicates that detection of a between‐conditions difference would have been possible with 17 participants (alpha = 0.05; power = 0.8; correlation among reported measures = 0.5; non‐sphericity correction = 1; G*power 3.1.9.7).

Compared with studies demonstrating positive effects of hypoxia on glycaemic control, our study differed in a number of ways that could account for the divergent findings. First, the beneficial effect of hypoxia on glycaemic control appears to be related to the magnitude of initial impairment (Lecoultre et al., 2013; Serebrovska et al., 2017); although being overweight is associated with impaired glucose homeostasis (Martinez et al., 2017; Teufel et al., 2021), our cohort had a normal glycaemic control and may not have been sufficiently metabolically compromised for any positive effects to manifest. Second, we employed a balanced cross‐over study design which included a control group undertaking a sham hypoxic exposure (CON); van Hulten et al. (2021) noted that most studies showing positive effects of hypoxia on glucose tolerance did not include an adequate control group. Finally, compared to studies utilizing repeated hypoxic exposures over multiple (10–14) nights (Lecoultre et al., 2013; Marlatt et al., 2020), as well as those employing severe hypobaric hypoxia (equivalent to 4300 m) *during* an OGTT (Kelly et al., 2010), the dose and timing of our hypoxic stimuli may have been insufficient to induce, or sustain, any effects on glycaemic control.

Although, a larger hypoxic stimulus might be necessary to affect glucose homeostasis over an acute timescale in individuals with overweight, our data suggest that reducing the F_I_O_2_ may not be feasible given that VHIGH was associated with increased dyspnoea and AMS symptoms, with two participants exhibiting AMS (Roach et al., 2018) and one participant withdrawn for exceeding the P_ET_O_2_ withdrawal criteria. However, brief intermittent exposures (e.g., Serebrovska et al., 2017), prolonged exposures to a more modest F_I_O_2_ (Lecoultre et al., 2013; Marlatt et al., 2020), or the inclusion of a period of acclimatization may enable a greater hypoxic stimulus whilst minimizing adverse symptomology. This may need to be balanced against the potential for elevated oxidative stress and inflammation; although this was not evident in our study, it has been reported in research employing a larger hypoxic stimulus (Siervo et al., 2014). Moreover, it is important to note that some studies have suggested that hypoxia may adversely impact on glycaemic control (Mackenzie & Watt, 2016), but this was not evident in our sample across the levels of hypoxia that we investigated.

Finally, our data indicated that whilst there was no between‐conditions difference in the total amount of energy derived from either carbohydrate or fat over the course of the OGTT, when these data were combined, a small, but statistically significant, decrease in total energy expenditure was evident in HIGH. Suppression of energy expenditure in the period following acute hypoxia has been reported previously by others in rodent (Mimura & Furuya, 1995) and human (Oltmanns et al., 2006) studies. In humans, the reduction was of a similar magnitude to the present study and was speculatively attributed to a reduced cerebral oxygen demand following hypoxia (Oltmanns et al., 2006). However, given that it is more generally accepted that metabolism is increased during acute hypoxia (Lippl et al., 2010), whilst the magnitude of decrease was similar to the coefficient of variation that has previously been reported for resting metabolic rate (Donahoo et al., 2004), we suggest that the physiological relevance of this observation in terms of energy balance across the entire experimental period is likely limited.

In conclusion, our balanced cross‐over pilot study demonstrated that acute exposure to simulated HIGH or VHIGH altitude prior to an OGTT did not influence glucose homeostasis, insulin sensitivity, markers of oxidative stress or inflammation, in males with overweight. However, VHIGH was associated with reduced tolerance of hypoxia, in terms of AMS symptoms, dyspnoea, and participant safety and therefore highlights feasibility challenges to using this dose in future studies. Future work should investigate: (i) the highest level of hypoxia that is reliably tolerated without adverse symptoms in individuals with overweight, (ii) the minimal effective exposure duration to this level of hypoxia in this cohort, and (iii) the recruitment of individuals with overweight with overtly compromised glycaemic control.

## ETHICS STATEMENT

Written informed consent was obtained before participation. The study was approved by the University's Faculty of Science and Health, Research Ethics Committee and conformed to the Declaration of Helsinki, except for registration in a database.
